# A Two-Tier Patient-Based Real-Time Quality Control Program Integrating K-Means Department Stratification and Inter-Instrument Ratios Improves Detection of Small Systematic Errors in Creatinine Testing

**DOI:** 10.3390/diagnostics16193238

**Published:** 2026-10-07

**Authors:** Xuemei Wei, Ziqing Jiang, Jingzhu Nan, Zefeng Yan, Zhu Lin, Rong Li, Jie Feng, Yanhong Gao

**Affiliations:** 1 Department of Clinical Laboratory, The First Medical Center of Chinese PLA General Hospital, Beijing 100853, China; 2 Department of Clinical Laboratory, Beijing Anzhen Hospital, Capital Medical University, Beijing 100029, China

**Keywords:** patient-based real-time quality control (PBRTQC), exponentially weighted moving average (EWMA), ratio method, K-means clustering, error detection, creatinine

## Abstract

**Background/Objectives:** Patient-based real-time quality control (PBRTQC) complements traditional internal quality control. This study validates a ratio-based PBRTQC integrating department clustering with inter-instrument ratio monitoring. **Methods:** Creatinine data (384,730 cases, 2025) from three measuring systems (A, B, C) were analyzed; conventional PBRTQC on unstratified Instrument A data served as comparator. Departments were stratified by K-means clustering of annual mean/SD, keeping the low-mean cluster (124 departments). The data of each measuring system were split chronologically: first-layer PBRTQC was trained on the first part, the second trained the ratio programs, and the third provided independent validation. Three systematic errors (fixed additive offsets of +5.28 and +10.56 μmol/L, and a progressive offset rising to +10.56 μmol/L, corresponding to 6% and 12% of the first-layer action limit) were simulated to assess the true positive rate (TPR), false positive rate (FPR), median samples to detection (MNPed), detection time and diagnostic odds ratio (DOR). **Results:** Under the fixed +5.28 μmol/L (6%) offset, A–B TPR was 28.83% versus 6.77% for traditional PBRTQC, with MNPed reduced from 34 to 20. Under the progressive offset rising to +10.56 μmol/L (12%), A–B TPR was 29.00% versus 8.35%, and detection time fell from 20 to 14 min. Under the fixed +10.56 μmol/L (12%) offset, A–B and A–C TPR reached 70.15% and 74.72% (MNPed 8 and 5; detection time 2 and 1 min). Across all scenarios the ratio programs achieved higher DOR. A stratified single-instrument comparator showed complementary contributions of stratification and ratio monitoring (TPR at the +5.28 μmol/L offset: 6.77%, 15.61% and 28.83%). **Conclusions:** Integrating department stratification with ratio-based PBRTQC markedly improves detection of small systematic errors in creatinine testing, albeit with more false positives, offering a transferable framework for laboratories using multiple same-platform measuring systems.

## 1. Introduction

Reliable detection of measurement errors in clinical laboratories is fundamental to ensuring patient safety. Traditional internal quality control (IQC) relies on periodic testing of commercial control materials to monitor measurement precision and accuracy, but it has inherent limitations: control materials exhibit matrix effects different from patient specimens, and suitable commercial controls are often difficult to obtain for low-concentration or special-matrix measurands [1,2,3].

In clinical laboratory quality management, patient-based real-time quality control (PBRTQC) serves as an important complementary approach. By statistically processing continuous patient test results, PBRTQC can supplement the fixed-time testing limitations of traditional control materials, providing continuous monitoring of measuring system performance and facilitating earlier detection of systematic errors. This method traces back to the “average of normals” proposed by Hoffmann and Waid in 1965 [4], and has since evolved through various algorithms—including the Bull algorithm for erythrocyte indices, moving average, moving median, and exponentially weighted moving average (EWMA) [5,6,7].

However, in practical application, particularly in laboratories of large general hospitals, PBRTQC performance is often constrained by a critical factor: patient population heterogeneity. In 2020, the International Federation of Clinical Chemistry and Laboratory Medicine (IFCC) Committee on Analytical Quality published recommendations for the performance verification of PBRTQC [8]. In 2024, the Chinese Society of Laboratory Medicine published the “Expert Consensus on the Procedure Establishment and Performance Verification of Patient-Based Real-Time Quality Control” [9], marking the formal entry of PBRTQC into standardized clinical application. The consensus explicitly identifies patient population heterogeneity as a key factor limiting the sensitivity of traditional single-instrument PBRTQC. Patients admitted to different clinical departments exhibit substantial differences in disease spectrum and physiological status, which directly leads to significant inter-departmental variations in the distribution of many test items, such as creatinine. For example, nephrology patients typically exhibit creatinine values concentrated in a higher range, whereas healthy individuals undergoing physical examination fall within the lower normal range. When data from these heterogeneous sources are combined for PBRTQC monitoring, this inherent biological variation substantially increases the overall dispersion of the data. The direct consequence is that control limits calculated from these mixed data become overly wide, rendering the PBRTQC system insensitive to small but clinically significant measurement drifts, thereby weakening its early warning capability—a phenomenon that is very common [10].

To overcome the interference caused by patient heterogeneity, researchers have recently begun exploring inter-instrument ratio-based PBRTQC methods [11]. The fundamental principle is that by calculating the ratio of results from the same department specimens tested simultaneously on two identical instruments, patient-specific biological variation can be partially cancelled out, making the ratio series primarily reflect measurement differences between the measuring systems. This approach has been preliminarily demonstrated to improve sensitivity for detecting small systematic errors. Nevertheless, existing studies have largely validated this method in the context of “whole-hospital data,” without adequately considering systematic differences between patient subgroups from different departments. A critical question remains: If data are first stratified by clinical department before implementing the ratio method, can more stable and precise quality control effects be obtained?

To address this gap, this study proposes and empirically validates a two-tier PBRTQC program. Departments were first stratified by K-means clustering of the annual mean and standard deviation of creatinine results, and the low-mean cluster was retained as a more homogeneous calibration population. The two-tier program then operates as follows: the first layer runs PBRTQC independently on each measuring system within the retained population, and the second layer constructs inter-instrument ratio sequences (A–B and A–C, with Instrument A as the target) from the first-layer outputs and monitors these ratios with independent PBRTQC programs. We hypothesize that, compared with traditional PBRTQC, this combined approach will significantly shorten alarm detection time, increase true positive rates, and explicitly quantifies the associated trade-off in false positive rates. To our knowledge, this is the first study to integrate department-level K-means stratification with inter-instrument ratio monitoring within a two-tier PBRTQC framework and to benchmark it against traditional single-instrument PBRTQC in a laboratory operating multiple identical measuring systems.

## 2. Materials and Methods

### 2.1. Data Source and Collection

**Figure 1:**
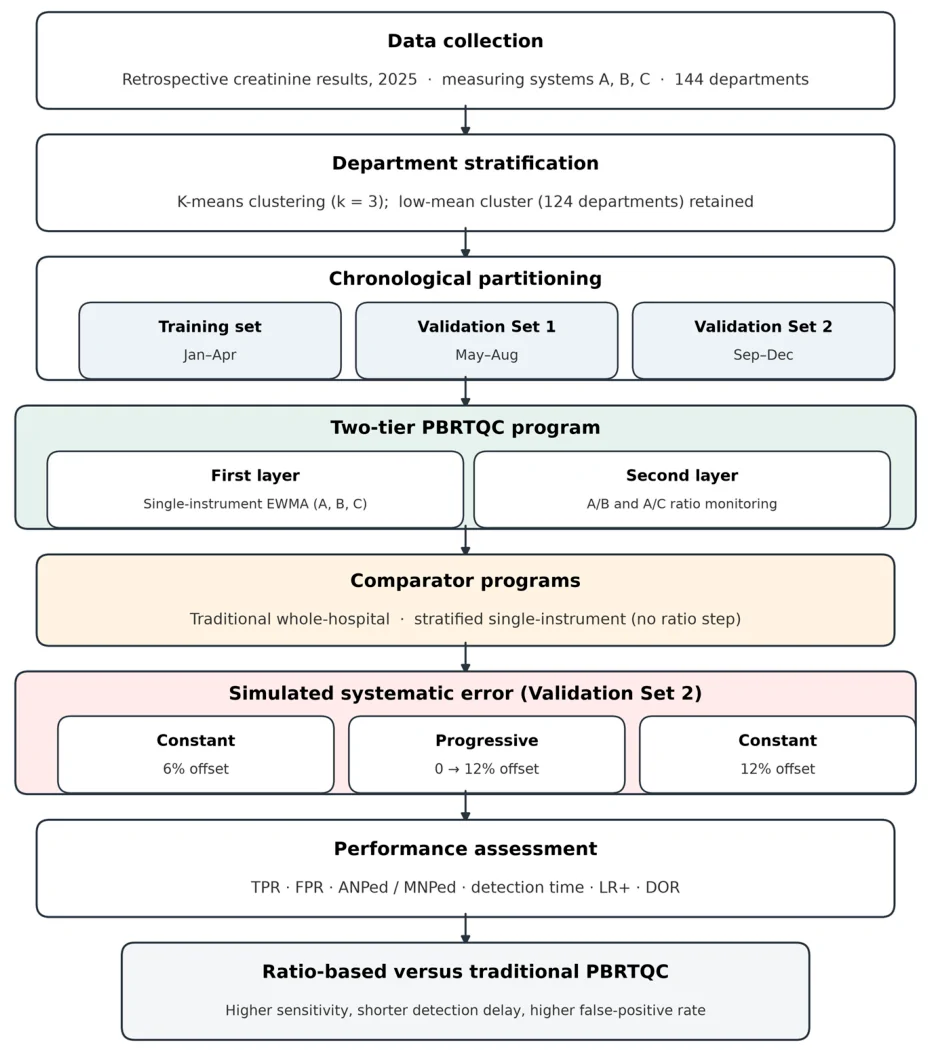
Workflow of the study. Creatinine results from three COBAS 8000 c702 measuring systems (A, B, C) were stratified by K-means clustering of the department-level mean and SD, and the low-mean cluster was retained. The data of each measuring system were partitioned chronologically into a training set, Validation Set 1 and Validation Set 2. The two-tier program comprises a first layer consisting of single-instrument EWMA PBRTQC applied independently to A, B and C, and a second layer monitoring the A/B and A/C ratio series derived from the paired first-layer statistics. Two comparator programs—the traditional whole-hospital PBRTQC and a stratified single-instrument program without the ratio step—were used to quantify the contribution of department stratification and of ratio monitoring. Systematic errors were then simulated on Instrument A in Validation Set 2, and performance was assessed with the true positive rate, false positive rate, average and median number of patient samples required for error detection, error detection time, positive likelihood ratio and diagnostic odds ratio. Arrows indicate the sequence of processing steps. EWMA, exponentially weighted moving average; WMA, weighted moving average.

This study retrospectively collected all creatinine (Cr) test data from the First Medical Center of Chinese PLA General Hospital in 2025 (1 January to 31 December), totaling 384,730 cases (Instrument A: 135,013 cases; Instrument B: 123,949 cases; Instrument C: 125,768 cases). All results were obtained from three Roche COBAS 8000 c702 biochemical measuring systems (Roche Diagnostics, Mannheim, Germany), designated as Instruments A, B, and C. The three instruments used the same lot numbers of reagents and calibrators and followed a unified daily IQC protocol. Data were extracted from the laboratory information system (LIS), including test date, instrument identifier, requesting department, patient ID, and creatinine test result. The technical workflow is shown in Figure 1.

All creatinine tests were performed using enzymatic colorimetric methods, consistent with the metrological traceability chain of each instrument. This study was conducted as a quality improvement evaluation using routine laboratory data and, according to institutional policy, did not require individual patient informed consent.

### 2.2. K-Means Clustering-Based Department Stratification

To reduce the impact of patient population heterogeneity on PBRTQC performance, clinical departments were stratified before PBRTQC calibration. Two statistics were calculated for each department: the mean (Mean) and standard deviation (SD) of annual creatinine test results. Departments with fewer than 1000 tests per year were excluded to ensure adequate statistical power; 46 departments were excluded by this criterion, leaving 144 departments for clustering.

K-means clustering is an unsupervised machine-learning technique and one of the most widely used clustering methods; it produces hard (each element belongs to a single cluster), flat, and polythetic (membership is determined by similarity based on multiple attributes) clusters [12]. Using the mean and SD as feature variables, K-means clustering was applied to all departments. The optimal number of clusters was determined by the Elbow Method combined with the Silhouette Score. The clustering identified three groups: a low-mean group (124 departments), an intermediate-mean group (16 departments), and a high-mean-high-SD group (4 departments). The high-mean-high-SD group comprised predominantly nephrology and intensive care departments, where creatinine results are typically elevated and highly variable, representing highly heterogeneous patient populations that do not meet the steady-state distribution assumption underlying PBRTQC methods. To construct a training population with minimal biological variation, only data from the low-mean group were retained for the ratio-based program; data from the intermediate-mean and high-mean-high-SD groups were excluded.

### 2.3. Data Partitioning

All creatinine data from the three measuring systems were divided by system identifier (Instrument A: 135,013 cases; Instrument B: 123,949 cases; Instrument C: 125,768 cases). The creatinine data from each instrument were chronologically ordered and divided into three equal parts: the training set (approximately January to April 2025), used to train the first-layer (single-instrument) PBRTQC parameters; Validation Set 1 (approximately May to August 2025), used to validate the first-layer parameters and to derive the second-layer ratio parameters; and Validation Set 2 (approximately September to December 2025), used as the fully independent validation set for evaluating the final ratio programs and their simulated error detection performance.

### 2.4. Ratio-Based PBRTQC Program Construction

Two ratio-based PBRTQC programs were constructed: the A–B ratio program and the A–C ratio program.

The program construction followed a two-tier, three-stage scheme corresponding to the three data partitions. In the first stage, the first-layer PBRTQC methods and parameters were trained on the training set (January to April 2025, restricted to the low-mean cluster departments), and the same configuration was applied independently to run PBRTQC on Instruments A, B, and C. In the second stage, Validation Set 1 (May to August 2025, likewise restricted to the low-mean cluster departments) was passed through the trained first-layer programs of the three instruments; this provided an operational check of the first-layer parameters and generated the first-layer PBRTQC statistic series. Using Instrument A as the target (reference) instrument, the statistic series from Instrument A was divided by the corresponding series from Instruments B and C to calculate the A-to-B ratio (A–B) and the A-to-C ratio (A–C), yielding two ratio time series. Because the three measuring systems process almost the same specimens nearly simultaneously, the paired first-layer statistics in the numerator and denominator were aligned in time and reflected virtually the same specimen stream, avoiding temporal mismatch. The theoretical expected value of the ratio is 1. When the target instrument (Instrument A) experiences a systematic shift while Instruments B and C remain stable, the corresponding ratio will deviate from 1. A second-layer PBRTQC algorithm was then run on each ratio sequence: candidate algorithms (weighted moving average [WMA] and EWMA) and their parameter combinations were evaluated, and the combination achieving the best trade-off between detection sensitivity and false alarms, under the constraint of an expected FPR ≤2% during this derivation phase, was selected as the final program. In the third stage, Validation Set 2 (September to December 2025) was likewise passed through the first-layer programs and converted into A–B and A–C ratio series; these series constituted the independent validation set on which the final ratio programs were evaluated (Section 2.7).

For the first layer, EWMA–Based PBRTQC was used, and the parameters were trained on the training set, yielding a common configuration applied identically to all three measuring systems (A, B, and C): a smoothing constant (weight) *λ* = 0.065, a window size *N* = 30, truncation by replacement within 0–142.4 μmol/L, a trigger point of 1 (a single limit violation suffices to raise an alarm), and no data transformation. The resulting target mean was 71.851 μmol/L and the SD was 5.082 μmol/L, with error/action limits of 58.287 and 88.000 μmol/L and warning limits of 62.809 and 82.617 μmol/L.

Throughout this work, five distinct quantities must be clearly distinguished: (i) the central tendency of the patient distribution (e.g., the first-layer target mean of 71.851 μmol/L); (ii) the biological (population) dispersion of individual patient results (SD ≈ 32 μmol/L in the low-mean cluster), which reflects case mix rather than measuring system performance; (iii) the statistical PBRTQC control limits, which are derived from the dispersion of the smoothed PBRTQC statistic itself (SD 5.082 μmol/L for the first-layer EWMA statistic) and not from the raw patient SD; (iv) the measurement performance specification, i.e., the total allowable error (TEa) of 12% for creatinine defined by WS/T 403-2024 [13]; and (v) the fixed additive systematic offsets used for validation, whose nominal 6% and 12% magnitudes are expressed relative to the first-layer upper action limit (88.0 μmol/L), corresponding to absolute offsets of +5.28 and +10.56 μmol/L (see Section 2.7). The SD of the patient distribution is therefore not interpreted as a permissible measurement error: the control limits are statistical constructs, and their clinical relevance is assessed against the TEa-referenced simulated errors.

### 2.5. Traditional PBRTQC Control

Using the whole-hospital creatinine dataset from Instrument A (all departments, without clustering-based stratification), a whole-hospital PBRTQC program (traditional PBRTQC) was established as the primary benchmark control. Its parameters were trained on the same training set (January to April 2025) under the same constraint of an expected training-set FPR ≤2%, and its error detection performance was assessed on Validation Set 2. It should be noted that, whereas the ratio programs were evaluated on low-mean-cluster data transformed through the first layer, the traditional program was evaluated on the unstratified whole-hospital data stream; this difference reflects the real-world setting each program represents and is acknowledged in the Limitations.

### 2.6. Stratified Single-Instrument Comparator Program

To quantify how much of the observed improvement is attributable to department stratification itself and how much to inter-instrument ratio monitoring, a stratified single-instrument comparator program was constructed. The comparator applied the first-layer configuration (EWMA, *λ* = 0.065, N = 30, truncation by replacement within 0–142.4 μmol/L) to the Instrument A data stream restricted to the low-mean cluster, without any inter-instrument ratio step. The program was implemented independently in Python (version 3.14) and validated against the QC-platform outputs on Validation Set 1 (identical mean of the smoothed statistic; 93.6% agreement in alarm classification). Because the independently implemented EWMA statistic is slightly smoother than the platform algorithm, its control limits were derived analogously to those of the first-layer program—target ± 2.12/1.78 SD for the warning limits and ± 3.18/2.67 SD for the action limits, estimated from the training-set statistic—which yielded realized false positive rates comparable to those of the other programs. As a sensitivity analysis, the comparator was also evaluated with the first-layer program’s official control limits. The same simulated error realizations as those used for the ratio programs were applied, and performance was evaluated with the identical metric definitions (Section 2.7).

### 2.7. Simulated Error Performance Assessment

Error introduction followed the Chinese health industry standard WS/T 403-2024 [13] “Analytical Performance Specifications for Common Clinical Chemistry Tests.” For creatinine, the total allowable error (TEa) is 12%. In Validation Set 2, only the data from Instrument A were subjected to three systematic positive offset error patterns, while Instruments B and C remained unchanged. All three patterns are fixed additive shifts applied to the affected results rather than proportional biases; their magnitudes are expressed as percentages of the first-layer upper action limit (88.0 μmol/L) to anchor the simulated errors to the statistical control limits shared by all evaluated programs:

(1) Fixed positive offset of +5.28 μmol/L: A constant additive offset equal to 6% of the first-layer upper action limit was inserted at a randomly selected time point each day and maintained until the end of that day in the Instrument A results.
(2) Progressive offset rising to +10.56 μmol/L: After a randomly selected time point each day, a linearly increasing additive offset was applied to Instrument A results, rising linearly from 0 at error onset to the full +10.56 μmol/L (12% of the action limit) at the end of that day.
(3) Fixed positive offset of +10.56 μmol/L: A constant additive offset equal to 12% of the first-layer upper action limit was inserted at a randomly selected time point each day and maintained until the end of that day in the Instrument A results.

The simulated data were input into the PBRTQC programs—the traditional whole-hospital program, the two ratio programs, and the stratified single-instrument comparator (Section 2.6) used for effect decomposition. For each combination of error pattern and program, the following performance metrics were calculated: true positive rate (TPR), false positive rate (FPR), false negative rate (FNR), ANPed and MNPed (average and median number of patient samples required for error detection), error detection time (mean and median, from error onset to first alarm, in minutes), likelihood ratios (LR+ and LR−), and diagnostic odds ratio (DOR). TPR was defined as the proportion of error-carrying samples (all samples from the daily insertion time point to the end of that day) that triggered an alarm; FNR equals 1 − TPR and is therefore not tabulated separately; and FPR was defined as the proportion of error-free samples that triggered an alarm; within this overall FPR, an alarm occurring within the effective smoothing span of the corresponding program after the last error-carrying sample (50 samples for the two-tier ratio programs (the 45-sample effective span of the first-layer EWMA plus the 5-sample second-layer window), 45 samples for the single-layer EWMA configuration with *λ* = 0.065, and 60 samples for the traditional program with λ = 0.049, corresponding in each case to a ≥95% decay of a unit inserted offset) was classified as a post-error residual alarm, and the remaining alarms at error-free samples were classified as baseline false alarms. ANPed and MNPed denote the average and median number of patient samples processed between error onset and the first alarm, and the error detection time was measured from error onset to the first alarm; these three quantities were computed within days carrying an inserted error. LR+, LR−, and DOR were derived from TPR and FPR as follows: LR+ = TPR/FPR, LR− = (1 − TPR)/(1 − FPR), and DOR = LR+/LR−.

### 2.8. Statistical Processing

Data processing and statistical computations were performed using R software (version 4.4, R Foundation for Statistical Computing, Vienna, Austria) [14] and Python (version 3.14). Clustering was implemented using the R “cluster” package [15] and “factoextra” package [16] for K-means algorithm execution and visualization. The PBRTQC calculation program was implemented in Python, and its software architecture was designed with reference to previously described PBRTQC monitoring platform implementations [17].

## 3. Results

### 3.1. Department Clustering and Stratification Results

**Figure 2:**
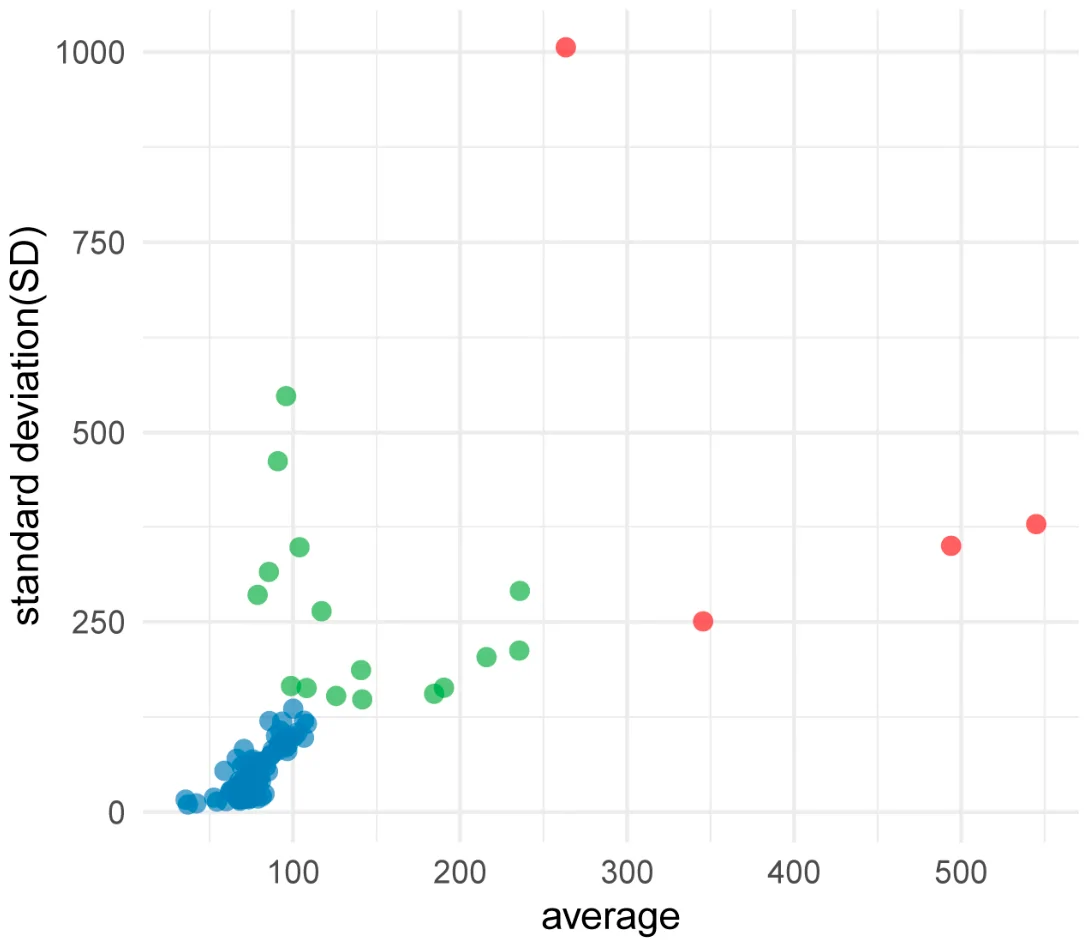
K-means clustering scatter plot of clinical departments (*k* = 3). The *x*-axis represents the annual mean creatinine of each department, and the *y*-axis represents the standard deviation. Different colors represent different clusters: red for the high-mean-high-SD group, green for the intermediate-mean group, and blue for the low-mean group.

This study ultimately included creatinine test data from 144 requesting departments. Using the mean (Mean) and standard deviation (SD) of historical test results from each department as clustering features, the K-means clustering algorithm was applied to stratify whole-hospital departments. By comparing the Silhouette Score and the Elbow Method (within-cluster sum of squares), the optimal number of clusters was determined to be 3 (*k* = 3). The characteristics of the three groups are shown in Table 1, and the scatter plot of departments in the (mean, standard deviation) feature space is shown in Figure 2. Of the three clusters, only the low-mean group (124 departments) was retained as the training population for the ratio-based program, whereas the intermediate-mean (16 departments) and high-mean-high-SD (4 departments) groups were excluded.

The pooled creatinine distribution of the retained low-mean cluster was additionally characterized to support the homogeneity assumption (Figure S1). In the training-period data of the three measuring systems (n = 113,262), the distribution was right-skewed, with a median of 69.4 μmol/L (interquartile range, IQR, 58.4–82.7 μmol/L; 2.5th–97.5th percentiles 40.1–115.2 μmol/L; mean 72.7, SD 32.0 μmol/L); the per-system distributions were nearly identical (medians 69.1, 69.6, and 69.8 μmol/L for A, B, and C, respectively; IQR widths 24.2–24.3 μmol/L). The relatively large SD therefore reflects the small proportion of high results in the right tail rather than a broad central dispersion: the central 50% of results span only 58.4–82.7 μmol/L, and the truncation limit of 142.4 μmol/L corresponds approximately to the 99th percentile of this population (99.0% of results lie below it). Because the first-layer EWMA statistic averages approximately 30 consecutive results, its dispersion (SD 5.082 μmol/L, approximately 7% of the target value) is far smaller than that of individual results, and the retained population therefore provides a suitably homogeneous monitoring basis.

To address the possibility that the clustering incorporated validation-period information, the clustering was repeated using training-period (January–April) data only. Relative to the stratification used in this study, low-mean cluster membership changed for only 7 of the 144 departments (binary agreement 95.1%), and these departments accounted for 0.5% of training-period samples; under an identical re-implementation, the whole-year and training-period partitions agreed for 93.8% of departments (Cohen’s κ = 0.70). Because K-means solutions depend on initialization, the department-level cluster assignments used in this study are available from the corresponding authors together with the raw data. The cluster structure is therefore stable and not materially dependent on validation-period information.

### 3.2. PBRTQC Parameter Training Results

The whole-hospital (traditional) program parameters were trained on the first one-third training set (training-set FPR constrained to ≤2%), and the first-layer PBRTQC parameters for the three instruments were likewise trained on this training set. The second-layer ratio parameters (A–B and A–C) were then derived on Validation Set 1 from the ratio series generated by the validated first-layer programs, under the same FPR constraint during derivation. On the independent validation set (Validation Set 2), the realized FPR was higher than this target (see Section 3.3), reflecting distributional differences between the parameter-derivation and validation periods; these values are reported transparently to enable a balanced assessment of sensitivity versus false alarms. The final parameters of each program are summarized in Table 2.

Inter-instrument comparability was assessed directly from the patient specimen stream rather than from the ratio outputs alone. During the error-free training period, the patient-result distributions of the three measuring systems were closely similar (means 72.49, 72.77, and 73.00 μmol/L; medians 69.0, 69.6, and 69.8 μmol/L; SD 32.25, 32.34, and 31.56 μmol/L for Instruments A, B, and C, respectively), and the same pattern was observed in Validation Set 1 (means 70.87, 71.80, and 72.28 μmol/L). The systematic component of the residual difference between Instrument A and the other two systems, expressed on the smoothed first-layer statistics, corresponded to a ratio of 0.9935 (A–B) and 0.9918 (A–C) in the training period and of 0.9896 (A–B) and 0.9838 (A–C) in Validation Set 1—that is, Instrument A read approximately 1.0–2.0% lower than Instruments B and C (bias −0.73 and −0.85 μmol/L in the training period; −0.96 and −1.39 μmol/L in Validation Set 1)—with a residual random scatter of SD 0.081–0.084 across both periods (2.5th–97.5th percentiles of the ratio 0.826–1.166) and limits of agreement of −13.1 to +11.2 μmol/L for the difference in smoothed statistics. These independently reproduced values agree closely with the second-layer parameters derived by the QC platform (targets 0.991 and 0.985, SD 0.084 and 0.086; Table 2). Because the second-layer programs monitor the ratio of the smoothed statistics of the same specimen stream, this small residual bias is absorbed by each program’s own target value and control limits rather than being assumed absent, while its random component defines the detection floor that the control limits must accommodate. External confirmation was obtained from the annual inter-measuring-system comparison required for ISO 15189 [18] laboratory accreditation: across 20 shared serum/plasma comparison samples (consensus means 24–2073 μmol/L), all 60 measurements from the three measuring systems passed the acceptance criteria, with individual biases ranging from −5.59% to +4.17% relative to the consensus means (mean biases +1.32%, −0.49%, and −0.83% for measuring systems A, B, and C, respectively) and mean pairwise differences of +1.85% (A–B) and +2.17% (A–C). These inter-system differences are well within the allowable total error (TEa) for creatinine (±12%, WS/T 403-2024) and are several-fold smaller than the simulated positive shifts (+5.28/+10.56 μmol/L, i.e., 6%/12% of the first-layer action limit) used to validate the programs (Table S3). Throughout the study period, the three systems used identical reagent and calibrator lot numbers and a unified daily internal quality control protocol (Section 2.1); detailed comparability statistics are provided in Tables S1 and S3.

### 3.3. Error Detection Performance

**Figure 3:**
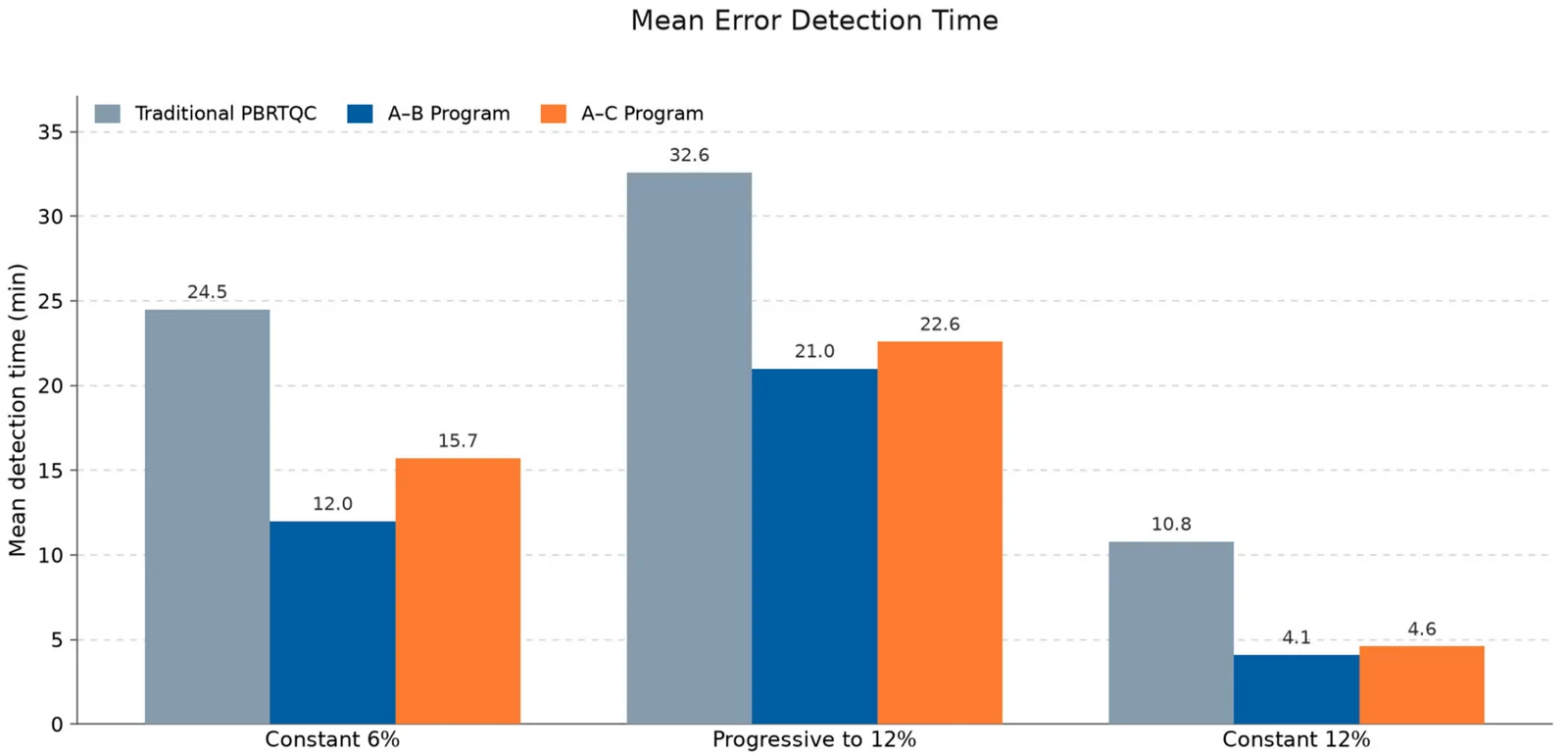
Comparison of error detection time (minutes) across three programs under three error modes. Lower values indicate faster detection. Bar charts display mean values.

**Figure 4:**
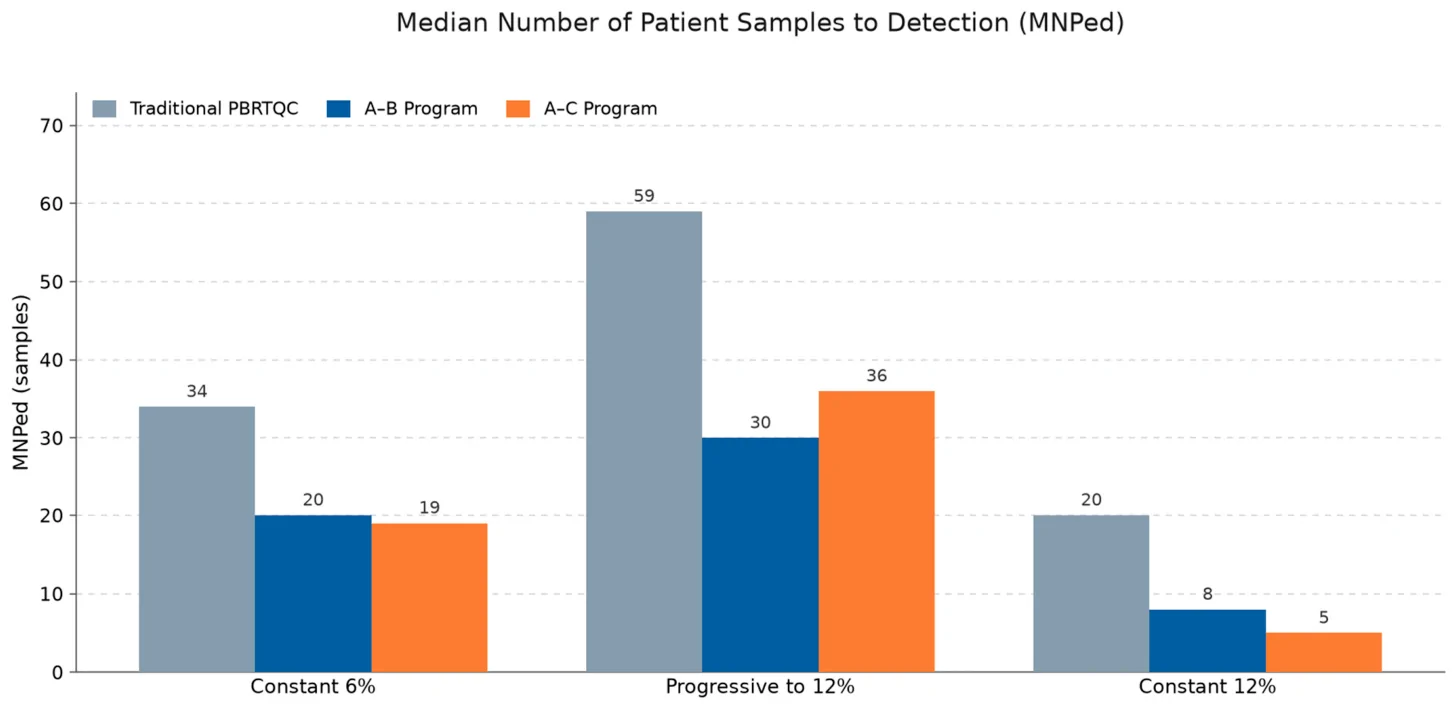
Comparison of MNPed across three programs under three error modes. Lower MNPed indicates faster error detection.

**Figure 5:**
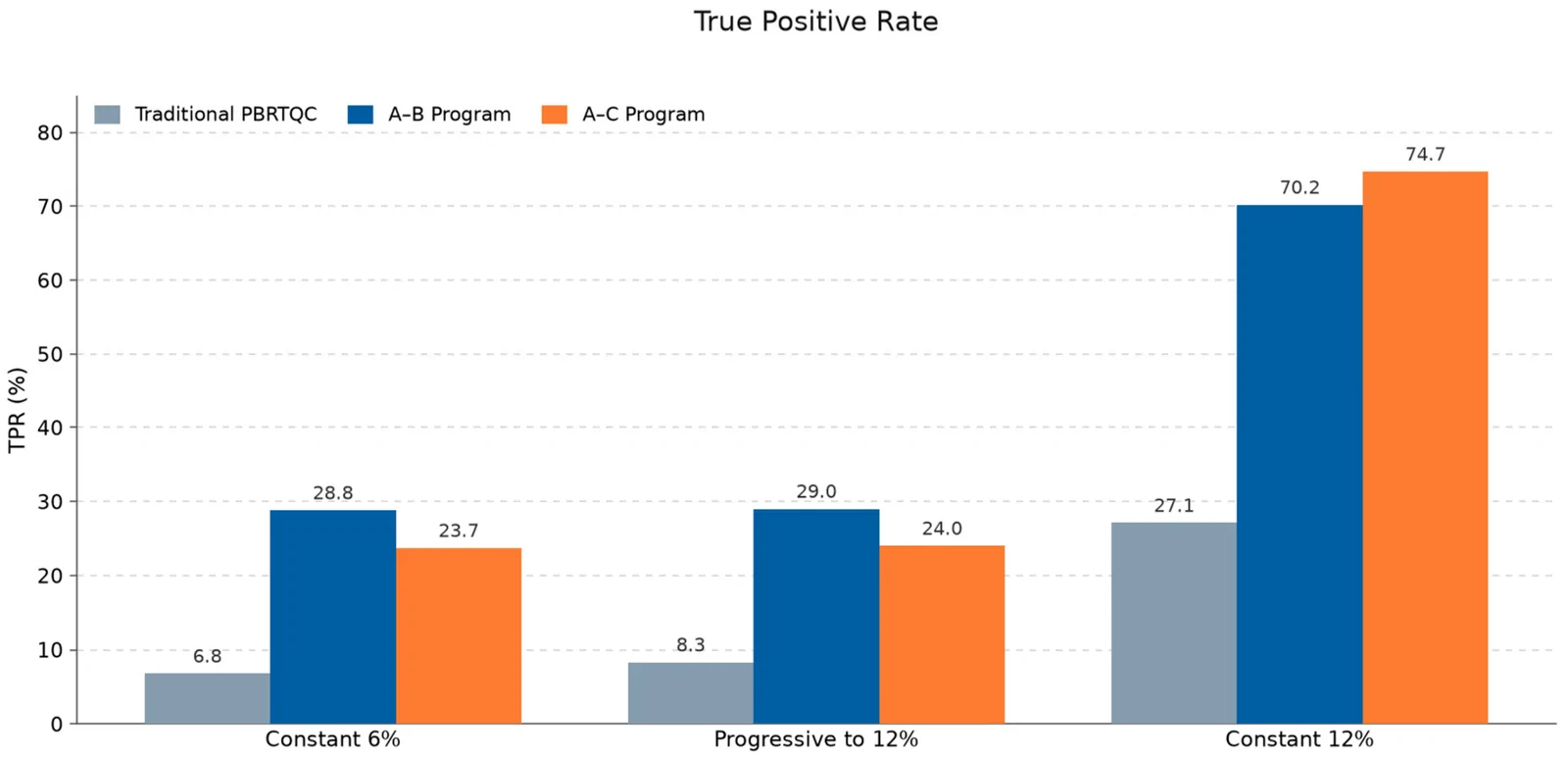
Comparison of true positive rate (%) across three programs under three error modes.

**Figure 6:**
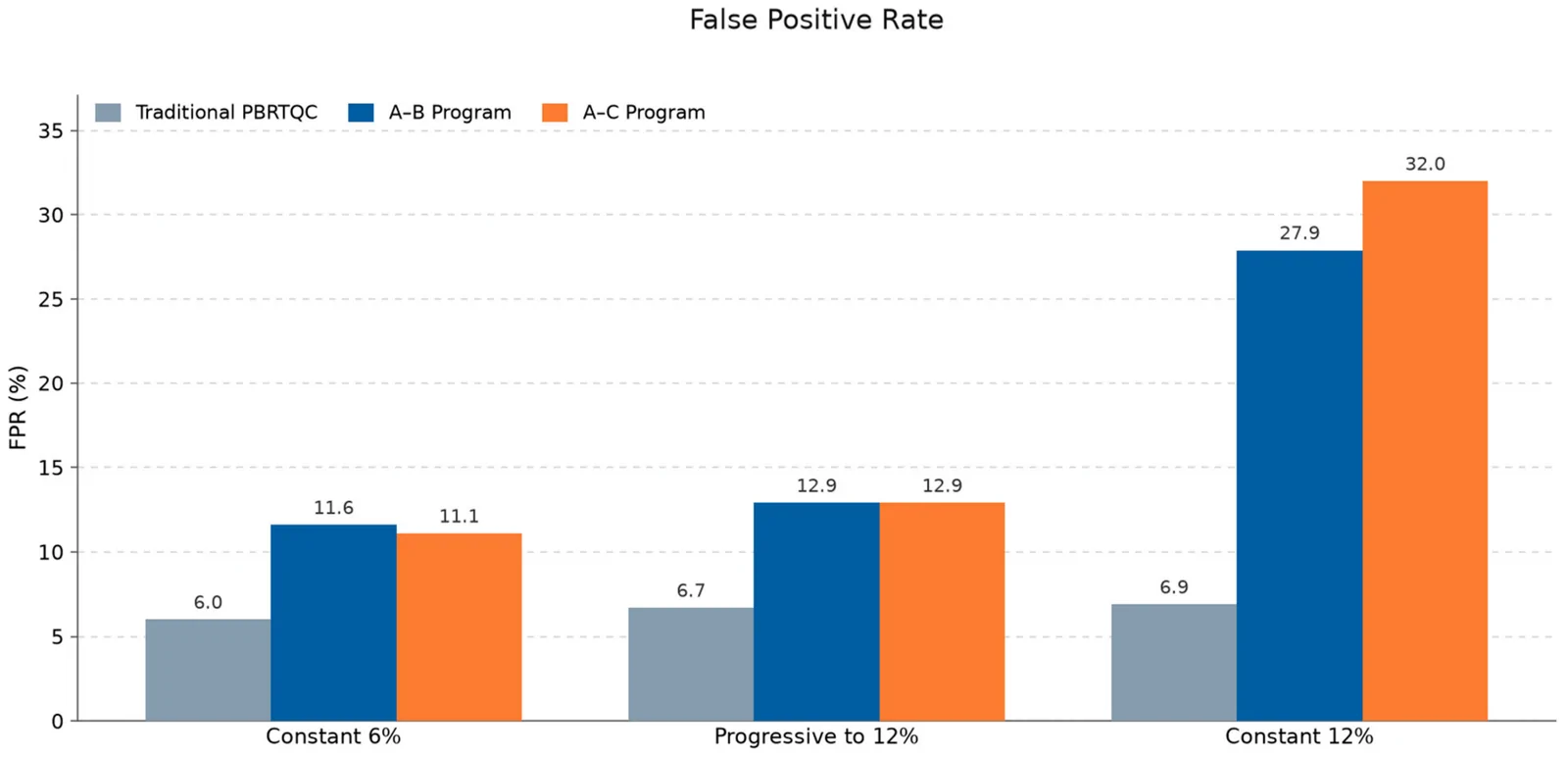
Comparison of false positive rate (%) across three programs under three error modes.

On Validation Set 2, performance was evaluated under three simulated error scenarios. Throughout validation, each program applied two-sided statistical control limits comprising a warning and an action (error) level (Table 2); the trigger point was set to 1, such that a single violation of either limit raised the corresponding alarm, and each alarm was classified as Warning or Error according to the limit pair exceeded. The comparative charts of error detection time, MNPed, true positive rate, and false positive rate for each error mode are shown in Figure 3, Figure 4, Figure 5 and Figure 6, respectively.

#### 3.3.1. Fixed Positive Offset of +5.28 μmol/L (6% of the Action Limit)

Under the fixed +5.28 μmol/L (6%) offset condition, the performance metrics are shown in Table 3 and Table 4. The traditional PBRTQC program achieved only a 6.77% TPR, with an MNPed of 34 and a median error detection time of 14 min, indicating extremely limited sensitivity at this error magnitude. In contrast, the A–B and A–C ratio programs increased TPR to 28.83% and 23.72%, respectively, representing 4.3-fold and 3.5-fold improvements. MNPed was reduced to 20 (A–B) and 19 (A–C), and median error detection times were shortened to 7 min and 6 min, respectively. In terms of diagnostic accuracy, the DOR values of A–B (3.09) and A–C (2.50) were substantially higher than that of traditional PBRTQC (1.13), indicating that the ratio programs possess superior discriminative efficacy for small systematic errors.

#### 3.3.2. Progressive Offset Rising to +10.56 μmol/L (12% of the Action Limit)

Under the progressive offset condition (rising linearly from 0 to +10.56 μmol/L), the performance metrics are shown in Table 5 and Table 6. The traditional PBRTQC program achieved a TPR of 8.35%, with an MNPed of 59 and a median error detection time of 20 min, reflecting insufficient responsiveness to trend-type errors. The A–B and A–C programs increased TPR to 29.00% and 23.99%, respectively, with MNPed reduced to 30 and 36. Median error detection times were shortened to 14 min and 16 min, and DOR values (A–B 2.76, A–C 2.13) were consistently higher than those of traditional PBRTQC (1.26).

#### 3.3.3. Fixed Positive Offset of +10.56 μmol/L (12% of the Action Limit)

Under the fixed +10.56 μmol/L (12%) offset condition, the differences in performance metrics among the three programs were most pronounced (Table 7 and Table 8). The traditional PBRTQC program achieved a TPR of 27.07%, with an MNPed of 20. The A–B and A–C programs achieved substantially higher TPR of 70.15% and 74.72%, respectively, with MNPed reduced to 8 and 5, average detection delays of 4.06 and 4.56 min, and median detection delays of 2 and 1 min. In terms of diagnostic efficacy, the DOR of the A–B and A–C programs (6.09 and 6.28) were higher than that of traditional PBRTQC (5.01). It should be noted that under the +10.56 μmol/L (12%) offset condition, the false positive rates of A–B and A–C increased to 27.85% and 32.00%, respectively, higher than traditional PBRTQC (6.90%), which is related to the additional random fluctuation introduced by ratio calculation. Between the two ratio programs, the A–C program showed advantages in TPR (74.72% vs. 70.15%) and DOR (6.28 vs. 6.09).

### 3.4. Contribution of Department Stratification Versus Inter-Instrument Ratio Monitoring

To separate the contribution of department stratification from that of inter-instrument ratio monitoring, the stratified single-instrument comparator program (Section 2.6) was evaluated on the same simulated error realizations as the ratio programs (Table S2). Under the +5.28 μmol/L (6%) offset, department stratification alone increased the TPR from 6.77% (traditional whole-hospital program) to 15.61%, while the ratio programs contributed the remainder of the gain (A–B, 28.83%; A–C, 23.72%); with these re-derived comparator limits, approximately half of the sensitivity improvement at this magnitude is thus attributable to stratification itself—an attribution that depends on the control-limit specification, as the same comparator operated with the official first-layer limits yielded a TPR of only 5.32% at this offset (see below). Conversely, the FPR of the stratified comparator (4.96%) was comparable to that of the traditional program (6.04%), whereas the FPR of the ratio programs was roughly twice as high (11.59% and 11.06%); the elevated false positive rate of the ratio design is therefore attributable to the ratio step rather than to department stratification. Under the progressive offset scenario, the TPR followed the same ordering (8.35%, 18.83%, and 29.00%/23.99% for the traditional, stratified, and ratio programs, respectively), although stratification alone did not shorten detection latency at this magnitude (MNPed 74 versus 59 samples), whereas the ratio step dominated the speed gain (MNPed 30/36). Under the +10.56 μmol/L (12%) offset, the stratified comparator alone reached a TPR of 61.03% (LR+ 11.12; DOR 26.96), approaching the ratio programs (70.15% and 74.72%), with stepwise shortening of MNPed (20 → 16 → 8/5 samples) and median detection time (6 → 5.9 → 2/1 min). The LR+/DOR of the stratified comparator were 3.15/3.55 and 3.20/3.71 under the 6% and progressive scenarios, respectively, lying between the traditional and ratio programs. When the comparator was instead operated with the first-layer program’s official control limits (sensitivity analysis; Section 2.6), its TPR was 5.32%, 7.14%, and 36.70% (FPR 1.84%, 2.34%, and 2.27%), and the qualitative conclusions were unchanged: the ratio step dominates detection speed and the increased alarm burden originates from the ratio step, whereas the share of the sensitivity gain attributable to stratification alone ranged from approximately half (re-derived limits) to little or no gain (official limits), indicating that this share is dependent on the control-limit specification.

### 3.5. Overall Performance Comparison

In terms of detection sensitivity and speed, the ratio programs outperformed the traditional PBRTQC program across all three error scenarios, whereas their false positive rates were higher throughout (Section 3.3). The sensitivity advantage was particularly pronounced at small error magnitudes: under the fixed +5.28 μmol/L (6%) offset, the ratio programs achieved 3.5- to 4.3-fold TPR improvements, with MNPed reduced by 41% to 44% and median error detection time shortened by 50% to 57%. Under the progressive drift scenario, ANPed was reduced by approximately 39% to 42%, and DOR increased by approximately 1.7- to 2.2-fold. At the maximum offset magnitude (+10.56 μmol/L), both ratio programs achieved TPR exceeding 70%, with median error detection time shortened to 1–2 min; at this magnitude, however, the traditional program retained a higher LR+ (3.92 vs. 2.52 and 2.34) owing to its substantially lower FPR, whereas the DOR of the ratio programs remained higher (6.09 and 6.28 vs. 5.01) because of their markedly lower LR−.

Comparative visualizations under the three error modes are shown in Figure 3, Figure 4, Figure 5 and Figure 6.

## 4. Discussion

This study demonstrates that the integration of department-level data stratification with inter-instrument ratio monitoring can substantially improve PBRTQC sensitivity for detecting systematic measurement errors, particularly for small constant offsets and progressive drifts that are most challenging to detect in routine clinical laboratory operation.

The core advance of this study lies in the establishment of a creatinine data-stratified ratio-based PBRTQC program. Traditional single-instrument PBRTQC trained on whole-hospital mixed data has a well-documented limitation: patient population heterogeneity increases the dispersion of quality control variables, leading to overly wide control limits. When data from nephrology patients (whose creatinine values often exceed the normal range by several-fold due to severe renal impairment) are mixed with data from general medical and surgical departments, the estimated standard deviation of the mixed distribution significantly exceeds that of any individual department. This increased data dispersion is the direct mechanistic reason for the extremely low sensitivity of traditional PBRTQC at the +5.28 μmol/L (6%) offset level (TPR 6.77%), consistent with findings from existing studies [10]. By restricting the training population to the 124 departments in the low-mean group, where patient creatinine values are predominantly within the normal to mildly elevated range, this study positioned the control limits to a reference population with smaller biological variation, thereby improving error detection sensitivity.

The second tier of this study—inter-instrument ratio monitoring—leverages the characteristics of a multi-measuring-system environment. The preceding department stratification has already selected patients with smaller biological variation. Taking the ratio of results from such patients tested on different instruments yields a signal that tracks only the measurement performance differences between the measuring systems. Under the condition that one instrument experiences systematic drift while the control instruments remain stable, this ratio will deviate from 1 in a manner proportional to the error magnitude. In the fixed +5.28 μmol/L (6%) offset scenario, this mechanism increased TPR from 6.77% to 28.83% (A–B), a 4.3-fold gain with direct practical significance. Decomposition using the stratified single-instrument comparator (Section 3.4) indicates that this gain arises from two complementary sources: department stratification alone raised the TPR at this offset from 6.77% to 15.61% without increasing the false positive rate, while the inter-instrument ratio step contributed the remainder and dominated the improvement in detection speed.

In this study, the traditional PBRTQC program employed a relatively low smoothing coefficient (*λ* = 0.049) and a large block size (N = 40). This parameter combination arises from the data characteristics it processes: the creatinine concentration of whole-hospital mixed patient data exhibits substantial inter-departmental differences, and biological variation contributes to high background noise. In the EWMA method, a lower λ value gives greater weight to historical means, which can suppress the interference of individual outlier values and control the false positive rate; however, this conservative strategy comes at the cost of delayed response to true systematic shifts. In contrast, the ratio programs (A–B, A–C) effectively eliminate inter-patient biological variation, with variation primarily originating from inter-instrument measurement errors, which have much lower dispersion. Against this low-noise background, a higher λ value (A–C program λ = 0.333) and a smaller block size (N = 5) make PBRTQC more efficient, enabling rapid tracking when inter-instrument ratios deviate, while balancing sensitivity and response speed. These parameter configurations are consistent with the recommended ranges for EWMA programs in the existing literature: λ for raw measurand sequences is typically recommended at 0.05–0.20 [8,19,20], and when the natural dispersion of the monitored variable is small, adopting values above this range is well-supported by statistical theory.

Under the fixed +10.56 μmol/L (12%) offset condition, the ratio programs exhibited elevated false positive rates (A–B: 27.85%; A–C: 32.00%, compared with 6.90% for traditional PBRTQC), and this result requires cautious interpretation, as the trade-off between detection sensitivity and false alarms remains a central topic of debate in PBRTQC implementation [21]. Several possible explanations exist for the marked increase in the false positive rates of the ratio programs under the 12% condition compared with smaller offsets. First, the ratio amplifies random fluctuations between instruments. Even when both instruments operate within normal ranges, small random differences exist. The ratio operation magnifies these differences, with the increased variance associated with elevated means generating some spurious high-value signals. Second, carry-over of the inserted errors likely contributes substantially: because the moving-average statistics contaminated by an inserted offset decay only gradually, windows shortly after error insertion (for example, early the following day before the new insertion time point) remain nominally error-free yet still partially reflect the preceding error, and larger offsets produce stronger contamination and thus more carry-over alarms, a pattern consistent with the monotonic increase in observed FPR across the three scenarios (Table S4). Third, inter-instrument common changes in the same direction but with different magnitudes may occur. For example, when reagent lot numbers are changed or ambient temperature drifts, all three instruments are typically affected in the same direction, but the response magnitude differs for each instrument. This situation can cause the ratio to temporarily elevate while neither instrument individually may have experienced a true systematic error. Fourth, the control limit setting may be insufficient. The current fixed control limits may not fully cover the amplitude of seasonal fluctuations during the independent validation period and remain to be optimized. Based on these observations, subsequent studies may consider introducing dynamic control limit schemes—that is, modeling the intra-day and inter-day variance structures so that control limits can adaptively adjust with data fluctuations. This approach is consistent with recommendations from previous studies [22,23,24]. From an operational perspective, an FPR approaching 30% at the 12% offset implies a substantial alarm burden that may be difficult to accept in routine practice and could erode operator trust—the classic “crying wolf” problem. The clinical and operational value of the ratio programs must therefore be weighed explicitly as a sensitivity–false alarm trade-off, ideally in combination with the alarm-management strategies discussed above.

K-means clustering based on (mean, SD) effectively separated high-creatinine departments from the remaining departments. This stratification step is conceptually consistent with the explicit recommendation in the 2024 Chinese PBRTQC Expert Consensus [9] that inter-departmental biological differences should be considered when calibrating PBRTQC programs. The excluded departments are characterized by kidney disease patients, whose creatinine results are systematically elevated and highly variable; including these departments in a unified PBRTQC training set would widen control limits and mask small shifts in the main patient population—precisely the problem that the clustering step is designed to address. The clustering method employed in this study is simple and practical, requiring only two routinely computable summary statistics, and is not limited to creatinine testing; the same method can be applied to any measurand with significant inter-departmental distribution heterogeneity. It may be argued that departments with clinically predictable distributions, such as nephrology or intensive care, could be excluded a priori on clinical grounds alone. The data-driven approach used here, however, additionally separates departments whose creatinine distributions are not predictable from their clinical names, sets the retained/excluded boundary objectively from the data rather than by expert judgment, and is therefore reproducible across laboratories; re-clustering restricted to training-period data reproduced 93.8% of the original stratification (Section 3.1), further supporting the robustness of this boundary.

This study has the following limitations. First, only a single measurand (creatinine) was included, and the choice of creatinine itself shapes the observed performance: its high test volume and marked inter-departmental heterogeneity provide a rigorous test of PBRTQC robustness under real-world conditions [25,26]; because the un-stratified comparator operates on this heterogeneous population, the benefit attributable to stratification itself is measurand-dependent and control-limit-dependent (Section 3.4) and would be smaller for measurands with narrower inter-departmental distributions, and whether this method has equivalent efficacy for other biochemical measurands with different distribution characteristics or measurement properties requires further validation [27]. Second, transferring this procedure to other laboratories depends on the availability of adequate computing resources and sufficiently large daily test volumes, because both the two-tier architecture and the simulation-based validation are computationally intensive and data-hungry. Third, Validation Set 2 corresponds to the most recent historical period in the dataset, and the temporal partitioning strategy may not have completely eliminated time-trend effects. Fourth, the elevated false positive rates of the ratio programs under large offsets (up to approximately 32%) imply a substantial alarm burden; the method should therefore be interpreted as a sensitivity–false alarm trade-off, and alarm-management strategies (e.g., trigger-rule tuning or dynamic control limits [22,23,24]) may be required before routine implementation. Fifth, performance metrics are reported as point estimates without confidence intervals, and no formal statistical hypothesis testing was performed for between-program comparisons; repeated simulations with confidence interval estimation would strengthen the statistical inference. Sixth, the department clustering was originally based on annual statistics and thus incorporated validation-period information; re-clustering restricted to training-period data changed low-mean cluster membership for only 7 of 144 departments, affecting 0.5% of training-period samples (Section 3.1), indicating that the cluster structure is not materially dependent on validation-period information.

The two-tier architecture described in this study is not inherently tied to creatinine or the specific algorithms employed. Extending to other biochemical measurands, immunoassay platforms, and settings with more than three instruments will help further validate its generalizability. In subsequent research, we will combine machine learning-based anomaly detection techniques [23] with ratio time series, which is expected to further enhance the program’s ability to capture anomalies under conditions where the data baseline itself is subject to fluctuation. As PBRTQC systems in modern laboratories evolve toward automated, algorithm-driven operational models [28], the low-mean group approach proposed in this study provides a conceptually clear and computationally practical foundation for multi-instrument quality monitoring.

## 5. Conclusions

This study proposes and validates a two-tier PBRTQC method based on inter-instrument ratios. The results demonstrate that, compared with traditional PBRTQC, the ratio-based PBRTQC programs (A–B and A–C) exhibit higher detection sensitivity under small-to-moderate systematic error conditions, with shorter detection delay times, offering practical advantages as a supplement to conventional quality control strategies. The ratio-based PBRTQC method is expected to serve as an effective supplementary tool for multi-instrument parallel quality control in large clinical laboratories and warrants further clinical validation before broader implementation.

The terminology used in this report follows the International Vocabulary of Metrology (VIM) [29] and recent recommendations on metrological terminology for laboratory medicine [30]; terms such as measurand, measurement accuracy, measurement precision, measuring system and measurement error are therefore used in their metrological sense, and “analysis” is reserved for statistical processing rather than for measurement.

## Figures and Tables

**Table 1:** K-means clustering results: cluster composition and characteristics.

Cluster	No. of Departments	Mean (μmol/L)	SD (μmol/L)	Patient Population Characteristics
Low-mean group	124	76.33	47.85	Patient distribution approximately within normal reference range; constitutes the main hospital population
Intermediate-mean group	16	140.53	254.09	Creatinine mean intermediate between high and low groups; includes urology and kidney-related departments
High-mean-high-SD group	4	411.93	496.26	Predominantly severe renal impairment patients; creatinine mean significantly above hospital-wide average

**Table 2:** Summary of optimized parameters for the three PBRTQC programs.

Program	Algorithm	*N* (Block Size)	*λ*	Target Value	SD	Error Upper	Error Lower	Warning Upper	Warning Lower
Traditional PBRTQC	EWMA	40	0.049	74.522 μmol/L	5.612 μmol/L	92.583	62.079	86.563	66.226
A–B Program (A–B Ratio)	WMA	5	—	0.991	0.084	1.201	0.782	1.131	0.851
A–C Program (A–C Ratio)	EWMA	5	0.333	0.985	0.086	1.199	0.771	1.128	0.842

Note: Traditional PBRTQC monitors the raw creatinine concentration of Instrument A. The A–B and A–C programs monitor inter-instrument ratio sequences; the theoretical target value is 1.0, and the actual target values and control limits were derived from the parameter-derivation data of each program (the training set for the traditional program; Validation Set 1 for the ratio programs). The “Error Upper” and “Error Lower” columns are action (error) limits, not truncation limits; truncation was by replacement within 0–124 μmol/L for the traditional program and within 0–142.4 μmol/L for the first-layer programs. WMA: Weighted Moving Average; EWMA: Exponentially Weighted Moving Average; “—“ indicates that WMA does not use the *λ* parameter. Creatinine concentrations are expressed in μmol/L; ratio values are dimensionless. The first-layer EWMA configuration (λ = 0.065, N = 30, target 71.851 μmol/L; Section 2.4) is a separate program from the traditional PBRTQC listed here. The target values in this table were derived from truncated, training- or derivation-period data and therefore differ from the annual department-level means reported in Table 1.

**Table 3:** Performance metrics under the fixed positive offset of +5.28 μmol/L (6%).

Program	TPR (%)	FPR (%)	ANPed	MNPed	Mean Error Detection Time (min)	Median Error Detection Time (min)
Traditional PBRTQC	6.77	6.04	51.74	34	24.51	14
A–B Program	28.83	11.59	26.09	20	12.02	7
A–C Program	23.72	11.06	32.14	19	15.68	6

Note: TPR, true positive rate; FPR, false positive rate; ANPed, average number of patient samples required for error detection; MNPed, median number of patient samples required for error detection; mean and median error detection time, from error onset to first alarm.

**Table 4:** Diagnostic efficacy metrics under the fixed positive offset of +5.28 μmol/L (6%).

Program	LR+	LR−	DOR
Traditional PBRTQC	1.12	0.99	1.13
A–B Program	2.49	0.80	3.09
A–C Program	2.14	0.86	2.50

Note: LR+ and LR−, likelihood ratios; DOR, diagnostic odds ratio. LR+ = TPR/FPR; LR− = (1 − TPR)/(1 − FPR); DOR = LR+/LR−.

**Table 5:** Performance metrics under the progressive offset rising to +10.56 μmol/L (12%).

Program	TPR (%)	FPR (%)	ANPed	MNPed	Mean Error Detection Time (min)	Median Error Detection Time (min)
Traditional PBRTQC	8.35	6.72	71.82	59	32.57	20
A–B Program	29.00	12.89	41.98	30	20.96	14
A–C Program	23.99	12.91	43.80	36	22.57	16

Note: TPR, true positive rate; FPR, false positive rate; ANPed, average number of patient samples required for error detection; MNPed, median number of patient samples required for error detection; mean and median error detection time, from error onset to first alarm.

**Table 6:** Diagnostic efficacy metrics under the progressive offset rising to +10.56 μmol/L (12%).

Program	LR+	LR−	DOR
Traditional PBRTQC	1.24	0.98	1.26
A–B Program	2.25	0.82	2.76
A–C Program	1.86	0.87	2.13

Note: LR+ and LR−, likelihood ratios; DOR, diagnostic odds ratio. LR+ = TPR/FPR; LR− = (1 − TPR)/(1 − FPR); DOR = LR+/LR−.

**Table 7:** Performance metrics under the fixed positive offset of +10.56 μmol/L (12%).

Program	TPR (%)	FPR (%)	ANPed	MNPed	Mean Error Detection Time (min)	Median Error Detection Time (min)
Traditional PBRTQC	27.07	6.90	25.02	20	10.78	6
A–B Program	70.15	27.85	12.05	8	4.06	2
A–C Program	74.72	32.00	10.40	5	4.56	1

Note: TPR, true positive rate; FPR, false positive rate; ANPed, average number of patient samples required for error detection; MNPed, median number of patient samples required for error detection; mean and median error detection time, from error onset to first alarm.

**Table 8:** Diagnostic efficacy metrics under the fixed positive offset of +10.56 μmol/L (12%).

Program	LR+	LR−	DOR
Traditional PBRTQC	3.92	0.78	5.01
A–B Program	2.52	0.41	6.09
A–C Program	2.34	0.37	6.28

Note: LR+ and LR−, likelihood ratios; DOR, diagnostic odds ratio. LR+ = TPR/FPR; LR− = (1 − TPR)/(1 − FPR); DOR = LR+/LR−.

## Data Availability

The data presented in this study are available on request from the corresponding author. The data are not publicly available due to privacy or ethical restrictions.

## Supplementary Materials

The following supporting information can be downloaded at: https://www.mdpi.com/article/10.3390/diagnostics16193238/s1, Figure S1. Distribution of creatinine results used for department stratification and of the retained low-mean cluster. (a) Pooled distributions of the three K-means clusters, computed on the whole-year department-level data (low-mean cluster, 124 departments; intermediate-mean, 16; high-mean/high-SD, 4). (b) Box plot of the retained low-mean cluster restricted to the three study measuring systems (A, B and C) and the training period (January–April 2025; n = 113,262). The dashed line indicates the truncation limit of 142.4 μmol/L, which corresponds approximately to the 99th percentile of this population (99.0% of results lie below it). In (b), the box spans the interquartile range, the horizontal line is the median, the red dot is the mean, and the whiskers extend to the 2.5th and 97.5th percentiles. Table S1. Inter-instrument comparability assessed directly from the patient specimen stream. Each ratio series is the ratio of the smoothed first-layer EWMA statistics of the same specimen stream (A/B and A/C), computed over error-free periods; n denotes the number of paired statistic values after exclusion of the 30-result warm-up window. Table S2. Performance of the stratified single-instrument comparator program compared with the traditional and ratio-based programs under the three simulated error scenarios. The comparator applies the first-layer configuration (EWMA, λ = 0.065, N = 30, truncation within 0–142.4 μmol/L) to Instrument A restricted to the low-mean cluster, without the ratio step. Table S3. Results of the 2025 annual inter-instrument comparison for creatinine (ISO 15189 accreditation programme). Table S4. Decomposition of the false positive rate (FPR) into baseline false alarms and post-error residual alarms.

## Abbreviations

The following abbreviations are used in this manuscript: ANPed, average number of patient samples needed for error detection; Cr, creatinine; DOR, diagnostic odds ratio; EWMA, exponentially weighted moving average; FNR, false negative rate; FPR, false positive rate; IQR, interquartile range; IQC, internal quality control; LIS, laboratory information system; LR+, positive likelihood ratio; LR−, negative likelihood ratio; MNPed, median number of patient samples needed for error detection; PBRTQC, patient-based real-time quality control; SD, standard deviation; TEa, total allowable error; TPR, true positive rate; WMA, weighted moving average.
