# Supplementary material for: A Two-Tier Patient-Based Real-Time Quality Control Program Integrating K-Means Department Stratification and Inter-Instrument Ratios Improves Detection of Small Systematic Errors in Creatinine Testing
Source: Diagnostics (Basel). 2026 Oct 7;16(19):3238. doi: 10.3390/diagnostics16193238 (PMC13664551; doi:10.3390/diagnostics16193238)
Supplement: Supplementary file 1 [file diagnostics-16-03238-s001.zip › diagnostics-4571193-supplementary.pdf]

Supplementary Materials

A Two-Tier Patient-Based Real-Time Quality Control Program Integrating K-Means Department Stratification and Inter-Instrument Ratios Improves Detection of Small Systematic Errors in Creatinine Testing

This file provides the supplementary material for the above manuscript: the distribution characteristics of the K-means clusters, the inter-instrument comparability assessed from the patient specimen stream, and the performance of the stratified single-instrument comparator program used to decompose the contribution of department stratification and inter-instrument ratio monitoring.

Figure S1. Distribution of creatinine results used for department stratification and of the retained low-mean cluster. (a) Pooled distributions of the three K-means clusters, computed on the whole-year department-level data (low-mean cluster, 124 departments; intermediate-mean, 16; high-mean/high-SD, 4). (b) Box plot of the retained low-mean cluster restricted to the three study measuring systems (A, B and C) and the training period (January–April 2025; n = 113,262). The dashed line indicates the truncation limit of 142.4 µmol/L, which corresponds approximately to the 99th percentile of this population (99.0% of results lie below it). In (b), the box spans the interquartile range, the horizontal line is the median, the red dot is the mean, and the whiskers extend to the 2.5th and 97.5th percentiles.

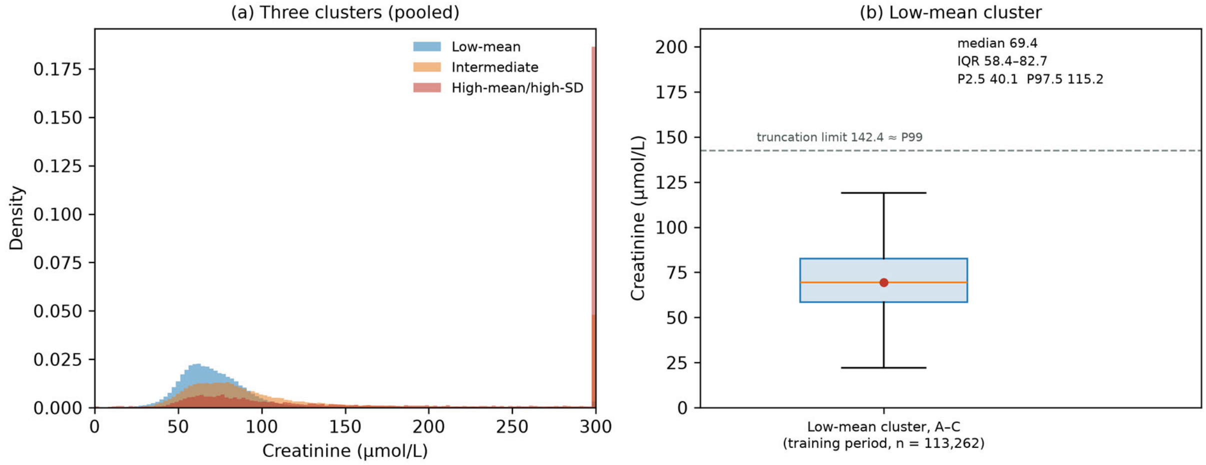

Table S1. Inter-instrument comparability assessed directly from the patient specimen stream. Each ratio series is the ratio of the smoothed first-layer EWMA statistics of the same specimen stream (A/B and A/C), computed over error-free periods; n denotes the number of paired statistic values after exclusion of the 30-result warm-up window.

**Pair**	**Period**	**n**	**Mean statistic A (µmol/L)**	**Mean statistic comparator (µmol/L)**	**Ratio mean**	**Ratio SD**	**Bias (µmol/L)**	**Bias (%)**	**95% limits of agreement (µmol/L)**
A-B	Training set (Jan–Apr)	33,840	70.87	71.60	0.9935	0.084	−0.73	−1.02	−12.6 to +11.2
A-C	Training set (Jan–Apr)	36,400	71.00	71.85	0.9918	0.084	−0.85	−1.18	−12.6 to +10.9
A-B	Validation Set 1 (May–Aug)	32,970	69.87	70.83	0.9896	0.081	−0.96	−1.35	−12.3 to +10.4
A-C	Validation Set 1 (May–Aug)	32,970	69.87	71.26	0.9838	0.084	−1.39	−1.96	−13.1 to +10.3

The 2.5th–97.5th percentiles of the ratio were 0.834–1.165 (A-B) and 0.838–1.166 (A-C) in the training period and 0.836–1.150 (A-B) and 0.826–1.157 (A-C) in Validation Set 1. The independently reproduced target and SD values agree closely with the second-layer parameters derived by the QC platform (A-B: target 0.991, SD 0.084; A-C: target 0.985, SD 0.086; Table 2 of the main text).

Table S2. Performance of the stratified single-instrument comparator program compared with the traditional and ratio-based programs under the three simulated error scenarios. The comparator applies the first-layer configuration (EWMA, λ = 0.065, N = 30, truncation within 0–142.4 μmol/L) to Instrument A restricted to the low-mean cluster, without the ratio step.

Scenario	Program	TPR (%)	FPR (%)	ANPed	MNPed	Mean detection time (min)	Median detection time (min)	LR+ / DOR
Constant 6%	Traditional PBRTQC	6.77	6.04	51.74	34	24.51	14	—
	Stratified single-instrument (self-consistent limits)	15.61	4.96	45.75	33.5	22.92	15.87	3.15 / 3.55
	A-B ratio program	28.83	11.59	26.09	20	12.02	7	—
	A-C ratio program	23.72	11.06	32.14	19	15.68	6	—
Progressive to 12%	Traditional PBRTQC	8.35	6.72	71.82	59	32.57	20	—
	Stratified single-instrument (self-consistent limits)	18.83	5.89	76.35	74	37.72	32.93	3.20 / 3.71
	A-B ratio program	29.00	12.89	41.98	30	20.96	14	—
	A-C ratio program	23.99	12.91	43.80	36	22.57	16	—
Constant 12%	Traditional PBRTQC	27.07	6.90	25.02	20	10.78	6	—
	Stratified single-instrument (self-consistent limits)	61.03	5.49	19.73	16	8.49	5.88	11.12 / 26.96
	A-B ratio program	70.15	27.85	12.05	8	4.06	2	—
	A-C ratio program	74.72	32.00	10.40	5	4.56	1	—
Sensitivity analysis	Stratified single-instrument, official first-layer limits: constant 6% / progressive / constant 12%	5.32 / 7.14 / 36.70	1.84 / 2.34 / 2.27	—	—	—	—	—

Table S3. Results of the 2025 annual inter-instrument comparison for creatinine (ISO 15189 accreditation programme).

Measuring system	Mean bias vs. consensus (%)	Bias range (%)	Samples passed
A	+1.32	−0.98 to +4.17	20/20
B	−0.49	−5.59 to +2.69	20/20
C	−0.83	−3.33 to +1.77	20/20
			placeholder

Consensus means are the means of the three measuring systems for each of the 20 shared serum/plasma comparison samples. Pairwise mean differences: A–B +1.85%, A–C +2.17%, B–C +0.37%. All 60 individual measurements were within ±6% of the consensus mean (59/60 within ±5%), i.e., within half of the allowable total error (TEa) for creatinine (±12%, WS/T 403-2024).

TPR, true positive rate; FPR, false positive rate; ANPed, average number of patient samples required for error detection; MNPed, median number of patient samples required for error detection; LR+, positive likelihood ratio; DOR, diagnostic odds ratio. Values for the traditional and ratio programs are reproduced from Tables 3–5 of the main text. "Self-consistent limits" denotes control limits derived from the comparator's own training-set statistic (± 2.12/1.78 SD for warning and ± 3.18/2.67 SD for action limits); the sensitivity analysis used the first-layer program's official control limits (target 71.851 µmol/L; action limits 58.287–88.000 µmol/L).

Table S4. Decomposition of the false positive rate (FPR) into baseline false alarms and post-error residual alarms.

Program	Metric	+5.28 µmol/L (6%)	Progressive to +10.56 µmol/L	+10.56 µmol/L (12%)
Traditional PBRTQC	Overall FPR (%)	6.04	6.72	6.90
	Baseline false-alarm rate (%)	5.36	5.36	5.36
	Baseline false alarms, n	860	860	860
	Post-error residual alarms, n	438	584	622
	Residual contribution (pp)	0.68	1.36	1.54
Stratified comparator†	Overall FPR (%)	4.96	5.89	5.49
	Baseline false alarms, n	688	658	776
	Post-error residual alarms, n	115	295	282
	Residual share of all false alarms (%)	14.3	31.0	26.7
A-B ratio program	Overall FPR (%)	11.59	12.89	27.85
	Baseline false alarms, n	1,242	1,242	3,050
	Post-error residual alarms, n	669	857	1,757
	Residual share of all false alarms (%)	35.0	40.8	36.6
A-C ratio program	Overall FPR (%)	11.06	12.91	32.00
	Baseline false alarms, n	1,273	1,273	3,719

	Post-error residual alarms, n	551	829	1,804
	Residual share of all false alarms (%)	30.2	39.4	32.7

Note. An alarm at an error-free sample was classified as a post-error residual alarm when it occurred within the effective smoothing span of the corresponding program after the last error-carrying sample (50 samples for the two-tier ratio programs, comprising the 45-sample effective span of the first-layer EWMA ($\lambda = 0.065$) plus the 5-sample second-layer window; 45 samples for the single-layer EWMA configurations with $\lambda = 0.065$; 60 samples for the traditional program with $\lambda = 0.049$), corresponding to a ≥95% decay of a unit inserted offset; all remaining alarms at error-free samples are baseline false alarms. For the traditional program, the baseline false-alarm rate was calculated over error-free samples outside the residual window, and the residual contribution (pp) equals the overall FPR minus this baseline rate. For the stratified single-instrument comparator†, baseline and residual alarms were classified by a true counterfactual re-run (identical configuration without inserted error); the counterfactual baseline false-alarm rates were 4.77%, 4.07%, and 4.49%, and the agreement between the window-based and counterfactual classifications was 98.8%, 97.5%, and 98.1% in the +5.28 µmol/L, progressive, and +10.56 µmol/L scenarios, respectively. pp, percentage points.
